# Comparison of electrically mediated and liposome-complexed plasmid DNA delivery to the skin

**DOI:** 10.1186/1479-0556-6-16

**Published:** 2008-12-04

**Authors:** Loree C Heller, Mark J Jaroszeski, Domenico Coppola, Richard Heller

**Affiliations:** 1 Center for Molecular Delivery, University of South Florida, Tampa, FL, USA; 2 Frank Reidy Research Center for Bioelectrics, Old Dominion University, Norfolk, VA, USA; 3 Department of Chemical Engineering, University of South Florida, Tampa, FL, USA; 4 Department of Oncologic Sciences, H. Lee Moffitt Cancer Center and Research Institute, Tampa, FL, USA; 5 Department of Molecular Medicine, University of South Florida, Tampa, FL, USA

## Abstract

**Background:**

Electroporation is an established technique for enhancing plasmid delivery to many tissues *in vivo*, including the skin. We have previously demonstrated efficient delivery of plasmid DNA to the skin utilizing a custom-built four-plate electrode. The experiments described here further evaluate cutaneous plasmid delivery using *in vivo *electroporation. Plasmid expression levels are compared to those after liposome mediated delivery.

**Methods:**

Enhanced electrically-mediated delivery, and less extensively, liposome complexed delivery, of a plasmid encoding the reporter luciferase was tested in rodent skin. Expression kinetics and tissue damage were explored as well as testing in a second rodent model.

**Results:**

Experiments confirm that electroporation alone is more effective in enhancing reporter gene expression than plasmid injection alone, plasmid conjugation with liposomes followed by injection, or than the combination of liposomes and electroporation. However, with two time courses of multiple electrically-mediated plasmid deliveries, neither the levels nor duration of transgene expression are significantly increased. Tissue damage may increase following a second treatment, no further damage is observed after a third treatment. When electroporation conditions utilized in a mouse model are tested in thicker rat skin, only higher field strengths or longer pulses were as effective in plasmid delivery.

**Conclusion:**

Electroporation enhances reporter plasmid delivery to the skin to a greater extent than the liposome conjugation method tested. Multiple deliveries do not necessarily result in higher or longer term expression. In addition, some impact on tissue integrity with respect to surface damage is observed. Pulsing conditions should be optimized for the model and for the expression profile desired.

## Background

The skin is an attractive target for gene therapy protocols for cutaneous diseases, vaccines and several metabolic disorders because it is easily accessible for both delivery and monitoring. To fully take advantage of skin as a target for gene transfer, it is important to establish an efficient and reproducible delivery system. Electroporation as a tool for the delivery of plasmid DNA is a strong candidate to meet these delivery criteria. Electroporation-mediated cutaneous plasmid DNA delivery has been demonstrated by many groups [1,2] for the eventual purpose of gene therapy.

Liposome or vesicle-complexed plasmid DNA has also been tested for enhancing transgene expression in the skin. Topical delivery has been performed in intact skin [3-9] and skin stripped of keratinocytes [10-12]. Intradermal injection of liposomes has been performed in a rat skin flap model [13]. This delivery may induce an immune response and has therefore been tested in vaccine delivery [8-11] and delivery has also been performed for therapeutic purposes [3,10,13]. In the study presented here, reporter expression was observed after intradermal injection of liposome-complexed DNA alone and in combination with *in vivo *electroporation.

Electroporation (EP) is a physical method that enhances delivery of molecules to tissues *in vivo*. Confined electrical pulses are delivered to tissues at levels which increase cell permeability without killing the cells, enabling molecules to pass through the cell membrane. EP has been used to effectively deliver chemotherapeutic agents to tumors in animals and in humans [14] and plasmid DNA to a variety of tissues in both animals and humans [1].

Recent studies have shown that electroporation efficiently delivers plasmid DNA to the skin resulting in increased local and serum expression levels compared to injection alone [15-31]. Skin electroporation delivery has been successfully performed in rodent [15,16,18-22,24,26-32], rabbit [25], pig [16,17,23,32] and non-human primate [16,32] model systems.

Several studies have been designed to use electrically mediated plasmid delivery for vaccine purposes, including hepatitis B surface antigen [17,22,23,25] and HIV [32]. Electrically mediated plasmid delivery to the skin has also been tested therapeutically. Delivery of the gene encoding erythropoietin to the skin achieved significantly elevated serum levels as well as significantly elevated hematocrit compared to injection of plasmid without EP [18]. Delivery of a plasmid encoding a growth factor [24,28] or a transcription factor which controls growth factor expression [31] increased wound healing. These studies demonstrate the feasibility of using this approach therapeutically or for increasing serum levels of a specific protein.

Molecule delivery is more efficient when the field is applied in more than one direction [33-35]. With two plate electrodes, the electrode must be repositioned for the second set of pulses. Therefore, a non-invasive four-plate electrode (4PE) was developed to allow the application of two sets of pulses rotated 90° with respect to each other, which makes pulse application more straightforward [29]. Delivery with this electrode results in reporter gene expression equivalent or superior to commercially available electrodes for delivery to the skin. The purpose of the experiments described here is to further investigate localized cutaneous plasmid delivery with the 4PE. Localized transgene expression levels and kinetics and histological damage were compared after the electrically mediated delivery of plasmid DNA. Delivery with the electrode was also tested in a larger rodent model, the rat.

## Methods

### Animals

Six to 7 week old female BALB/c mice (NCI) or 200–250 gram male Sprague Dawley rats were anesthetized in an induction chamber charged with 3% isoflurane in O_2 _then fitted with a standard rodent mask and kept under general anesthesia during treatment.

### Plasmid delivery

gWizLuc was commercially prepared (Aldevron, Fargo, ND). Endotoxin levels were < 0.1 EU/μg plasmid. For *in vivo *electroporation, 50 μl gWizLuc suspended to 2 μg/μl in sterile injectable saline was injected intradermally. Using a 4PE electrode [29], eight 100 V/cm 150 ms pulses at a frequency of 1 Hz were immediately applied with a BTX 830 pulse generator (BTX Molecular Delivery Systems, Holliston, MA) unless otherwise noted. For liposome delivery, 100 μg gWizLuc was complexed with a commercial preparation of DOTAP (*N*-[1-(2,3-dioleoyloxy) propyl]-*N,N,N*-trimethyl-ammonium-methyl-sulfate, (Roche Diagnostics, Mannheim, Germany) in a ratio of 1:1.6 (w/w) [10] and 50 μl was injected intradermally.

### Luciferase reporter assay

At the indicated time points after plasmid delivery, luciferase activity was quantified as previously described [36]. The treated area was consistently 6 mm in diameter. However, since there was some variation in the diametric tissue excised, activity was expressed in total ng luciferase per treatment area. Values represent mean and standard error. Experiments containing only two groups were analyzed by Student's unpaired T test. Experiments with greater than two groups were analyzed by nonparametric ANOVA.

### Histological analysis

For histological analysis, 50 μl 2 μg/μl gWizLuc was delivered using eight 150 ms 100 V/cm pulses with the 4PE. At the time points indicated, the mice were euthanized and a seven mm diameter circle of skin 2–3 mm thick that encompassed the 6 mm diameter treatment area was removed. After fixation in 10% neutral buffered formalin for six hours, each sample was dehydrated in ascending grades of ethanol, cleared in xylene, and infiltrated with paraffin. Following embedding, tissues were cut into four 4 mm sections. Sections were stained with hematoxylin and eosin and then examined histologically for damage. Samples were graded using a schema including surface damage, inflammation, bullae, muscle degeneration and subepidermal necrosis in eight 4 × 7 mm sections [29]. For surface damage, the percentage of each section damaged was determined. For the other damage assessments, any damage seen within a low power field (40×), even focal points, was considered positive. The percentage reported was the number of positive fields seen (eight fields per section and four sections per sample). The total amount of damage was determined for each sample and expressed as the mean and SEM of the percentage of the total treatment area. Significance was determined for the three groups by nonparametric ANOVA.

## Results and discussion

**Figure 1:**
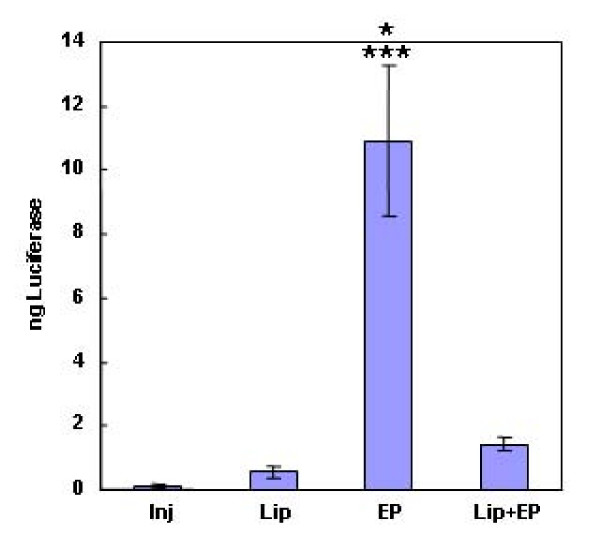
**Comparison of liposome and EP delivery of plasmid DNA**. Luciferase expression in mouse skin 48 hours after delivery of 100 μg gWizLuc as described in materials and methods. Inj, injection only, n = 12; Lip, liposomes, n = 12; Electroporation, EP, n = 12; Lip+EP, liposomes + EP, n = 4. ***p < 0.001 with respect to injection only; *p < 0.05 with respect to liposomes.

EP delivery previously optimized in mouse skin was directly compared to liposome-based delivery (Figure 1). At 48 hours, the DNA:DOTAP formulation tested tended to increase reporter expression. EP increased expression significantly, nearly 20 fold higher than the liposome formulation. When EP and liposome delivery were combined, expression was not significantly higher than injection alone. The combination of liposome delivery and *in vivo *electroporation for plasmid delivery has been compared in previous studies. Wells, *et al*. found no difference in transgene expression after delivery of a luciferase encoding plasmid by electroporation with six 1 ms 800–1600 V/cm pulses, with small unilamellar DOTAP lipoplexes, or with the combination to MC2 mouse mammary tumors [37]. Cemazar, et al. found that transfection efficiency of a plasmid encoding green fluorescent protein was more effective in complex with lipofectin than naked plasmid DNA injection when delivered to several mouse tumor types. However, electrically mediated delivery of plasmid alone using eight 5 ms 600 V/cm pulses significantly increased transfection. The combination of complexed DNA and electroporation was not significantly different from electroporation alone [38].

There are several possible reasons as to why the results of these three studies differ considerably. There are variations the lipid composition, the reporter gene delivered, the *in vivo *electroporation parameters, and the method of analysis (overall transgene expression vs. transfection efficiency). In addition, these studies used a tumor, rather than skin, model. Tumor cells typically divide more rapidly than skin cells. It is understood that both EP and liposomes can destabilize cell membranes and, in this particular case, perhaps the combination of the two is disruptive, leading to decreased cell survival and ultimately decreased expression. Alternatively, exposure of the liposomes to EP may release the DNA prior to contact with the membranes and reduce the transport of plasmid through the cell membrane which would also lead to reduced transgene expression.

Clearly, EP enhances skin expression after intradermal injection of plasmid DNA [15-23,25,26,29]. After a single cutaneous delivery, significantly increased reporter expression has been demonstrated in rabbits for two days [25], in mice [27] and rats [18,19,26] to seven days, and in mice to approximately two weeks [22,29]. The differences in levels and duration of expression may be due to the different models, plasmid constructs, electrodes, electroporation protocols, and methods of analysis used.

**Figure 2:**
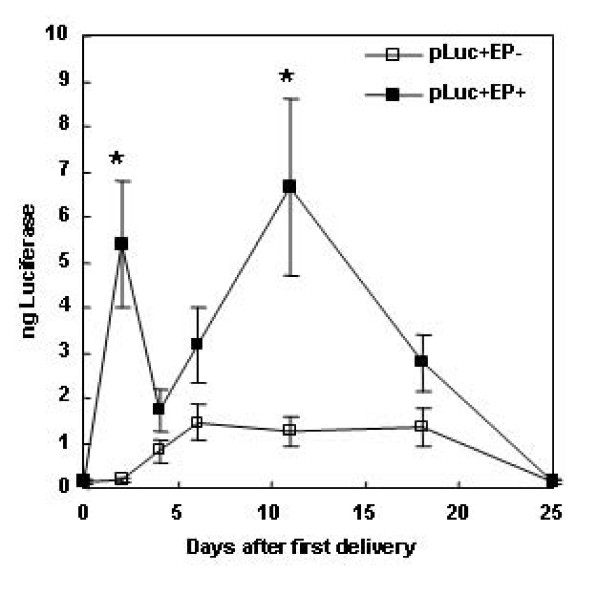
**Duration and levels of skin luciferase expression after delivery of plasmid by EP on days 0, 2, and 4**. Luciferase expression in mouse skin after delivery of 100 μg gWizLuc at days 0, 2, 4, 6, 11, 18, and 26 as described in materials and methods. n = 12. *p < 0.05 with respect to injection only at the specified time point.

**Figure 3:**
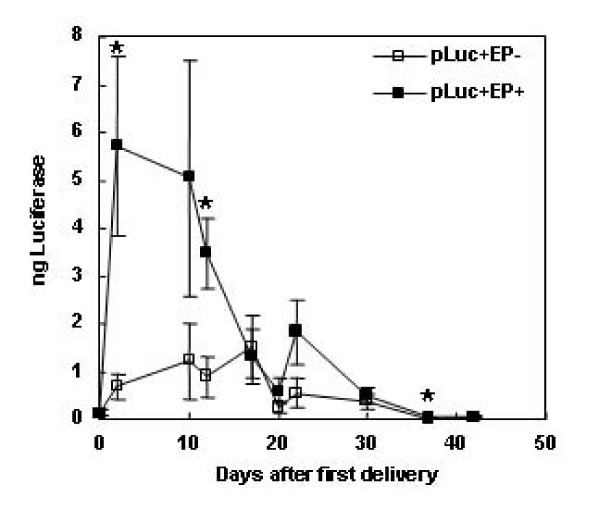
**Duration and levels of skin luciferase expression after delivery of plasmid by EP on days 0, 10, and 20**. Luciferase expression in mouse skin after delivery of 100 μg gWizLuc at days 0, 2, 10, 12, 17, 20, 22, 30, 37, and 42 as described in materials and methods. n = 12. *p < 0.05 with respect to injection only at the specified time point.

In an attempt to increase the duration of transgene expression, multiple deliveries were performed. Two delivery time courses were tested, day 0 followed by days 2 and 4 (Figure 2), and day 0 followed by days 10 and 20 (Figure 3). With deliveries at days 0, 2, and 4 (Figure 2), expression spiked 48 hours after the first delivery at 5.4 ± 1.4 total ng luciferase, similar to the levels observed previously [29]. This expression significantly decreased on days 4 and 6. Expression significantly peaked again at day 11 at 6.6 ± 1.9 total ng luciferase. This is possibly related to when the skin tissue recovered from any damage produced by the EP process. Multiple deliveries did not significantly increase the duration of transgene expression. This agrees with Lin, *et al*., who observed that two deliveries 24 hours apart did not result in increased luciferase expression [28] in a rat model.

When deliveries were performed on days 0, 10, 20, a similar immediate increase in reporter expression to 5.7 ± 1.9 total ng luciferase was observed (Figure 3). Interestingly, no spike in expression was observed after the day 10 delivery. However, day 12 expression was significantly higher than injection alone. A small but insignificant spike in expression was observed 48 hours after the day 20 delivery, and a statistically significant difference was also observed at day 37. Similar to the first time course, these multiple deliveries did not significantly alter the time course of reporter expression from that of a single plasmid delivery [29].

In this study, only a small increase in expression duration is observed with either multiple treatment protocol. Skin cell turnover time for mice is approximately 7–12 days. If tissue trauma due to EP were limiting expression, cell turnover should allow subsequent peaks in expression. Cell turnover may not facilitate increased expression with the short interval delivery, but with the second delivery time course, one would expect long-term increased expression especially following delivery at 20 days. For these reasons, histological analysis of the delivery site was performed (Table 1, Table 2). For both of these experiments, the second and third deliveries were performed at the same specific site as the initial delivery, since repeat procedures at the same site might negatively impact cellular integrity and reduce expression.

**Table 1:** Tissue damage after three deliveries on days 0, 2, and 4.

	**DNA+EP-**	**DNA-EP+**	**DNA+EP+**
**Surface Damage**			

**Day 4**	1.3 ± 0.5	14.2 ± 4.4**	9.5 ± 3.5
**Day 6**	2.4 ± 1.9	11.0 ± 3.0**	10.7 ± 3.0*

**Inflammation**			

**Day 4**	68.1 ± 12.7	100 ± 0	75.0 ± 11.5
**Day 6**	66.7 ± 11.2	95.8 ± 4.2	59.7 ± 10.3

**Bullae**			

**Day 4**	4.2 ± 4.2	47.2 ± 11.2*	43.1 ± 13.7
**Day 6**	4.2 ± 4.2	37.5 ± 6.5*	52.7 ± 13.1**

**Muscle Degeneration**			

**Day 4**	59.7 ± 12.0	97.9 ± 2.1*	72.2 ± 11.3
**Day 6**	66.7 ± 9.4	95.8 ± 4.2	52.8 ± 11.6

**Subepidermal Necrosis**			

**Day 4**	8.3 ± 5.6	48.6 ± 10.7*	43.8 ± 13.8
**Day 6**	12.5 ± 9.0	50.0 ± 10.7*	40.3 ± 13.5

Values represent the mean and SEM for three independent experiments (n = 4) for a total of 12 samples.

DNA, gWizLuc

EP, electroporation

**p < 0.01

*p < 0.05

**Table 2:** Tissue damage after three deliveries on days 0, 10, and 20.

	**DNA+EP-**	**DNA-EP+**	**DNA+EP+**
**Surface Damage**			

**Day 12**	2.4 ± 0.9	0.5 ± 0.2	3.0 ± 1.2
**Day 22**	3.8 ± 1.1	0.3 ± 0.2*	3.0 ± 1.1

**Inflammation**			

**Day 12**	68.0 ± 12.7	68.0 ± 9.3	95.8 ± 4.2
**Day 22**	52.6 ± 11.8	95.8 ± 4.2**	77.8 ± 6.9

**Bullae**			

**Day 12**	31.9 ± 14.1	8.3 ± 5.6	29.2 ± 13.2
**Day 22**	12.7 ± 5.9	4.2 ± 4.2	23.6 ± 9.5

**Muscle Degeneration**			

**Day 12**	68.0 ± 12.7	48.6 ± 10.7	84.7 ± 9.0
**Day 22**	55.1 ± 11.2	66.7 ± 11.2	79.2 ± 7.7

**Subepidermal Necrosis**			

**Day 12**	23.6 ± 12.88	12.5 ± 8.5	38.9 ± 14.1
**Day 22**	25.9 ± 8.8	4.2 ± 4.2	16.7 ± 9.4

Values represent the mean and SEM for three independent experiments (n = 4) for a total of 12 samples.

DNA, gWizLuc

EP, electroporation

**p < 0.01

*p < 0.05

After deliveries on days 0, 2, and 4, EP significantly increased surface damage, bullae, and subdermal necrosis over plasmid injection alone at both 4 and 6 days (Table 1). This damage may be ameliorated by the presence of plasmid DNA. No delivery type significantly increased inflammation more than any other. At day 4, muscle degeneration was increased significantly over plasmid injection alone, but this degeneration was resolved by day 6.

In the longer time course, deliveries were performed at days 0, 10, and 20, while histological analysis was performed at days 12 and 22 (Table 2). In this time course, low levels of surface damage were observed, although a significant increase with EP alone was observed over plasmid injection alone at day 22. Inflammation was also increased with EP alone at this time point. No significant differences were observed between delivery types in bullae, muscle degeneration, or subepidermal necrosis.

Although damage is observed in each time course following the second delivery, this damage does not increase after the third delivery. While there were no obvious safety issues with repeat deliveries, the results presented here suggest that repeat administrations should not be performed at the same specific site.

**Figure 4:**
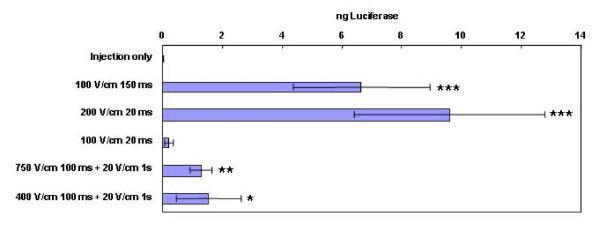
**EP delivery of plasmid DNA to rat skin**. Luciferase expression in skin 48 hours after delivery of 100 μg gWizLuc with eight pulses at the conditions noted. n = 12. ***p < 0.001 with respect to injection only; **p < 0.01 with respect to injection only; *p < 0.05 with respect to injection only.

It was important to demonstrate that plasmid delivery with the 4PE would increase skin transgene expression in a larger model with thicker skin, the rat. Pulses of 100 V/cm and 150 ms resulted in the highest expression levels in the mouse [29]. In mouse skin, 20 ms pulses of 100 or 200 V/cm pulses increased expression to approximately 60% of 100 V/cm 150 ms. However, in the study described here, while several pulse types resulted in significantly higher reporter expression (Figure 4), a longer or higher field strength pulse was necessary for the higher levels of expression rat skin. This may reflect the differences in skin architecture between the two models. While many EP protocols may increase transgene expression in multiple models, some protocol optimization is necessary based on skin structure and thickness.

## Conclusion

Electroporation is an effective method for *in vivo *delivery of plasmid DNA [1,2], and this approach is also an effective tool for cutaneous applications. As has been seen with other tissues, a variety of EP protocols ranging from short, high field strength to long, low field strength pulses as well as both invasive and surface electrodes have been tested. When developing a protocol for a skin based application, it is important to consider all of these variables as each will contribute to the levels and duration of expression obtained. Expression levels in response to delivery of plasmids encoding potentially toxic molecules such as cytokines should be tightly controlled with only short term expression. Expression after delivery of plasmids encoding potential vaccine candidates also may only require short-term expression. However, in the case of replacement of a defective protein such as Factor IX, long-term expression is desirable.

The results obtained in the current study demonstrated that a non-invasive surface electrode can be used to deliver plasmid DNA to the skin. Delivery was successful in both mice and rats. The highest expression levels in each species were obtained with different EP parameters. While this delivery method is safe, if an application is being developed that requires multiple administrations, it is advisable to not perform the repeat at the same exact site as the first administration. As has been seen with delivery to other tissues, EP is a safe and reliable method to obtain efficient and effective delivery of plasmid DNA.

## Abbreviations

DNA: deoxyribonucleic acid, or gWizLuc specifically in Tables 1 and 2; EP: electroporation; 4PE: four-plate electrode; DOTAP: (*N*-[1-(2,3-dioleoyloxy) propyl]-*N,N,N*-trimethyl-ammonium-methyl-sulfate.

## Competing interests

With respect to duality of interest, Drs. Richard Heller and Jaroszeski are co-inventors on patents which cover the technology that was used in the work reported in this manuscript. The patents have been licensed to RMR Technologies, LLC and sublicensed to Inovio biomedical Corporation. Both Drs. Richard Heller and Jaroszeski have ownership interest in RMR Technologies and own stock and stock options in Inovio.

## Authors' contributions

LH was involved in the experimental work, data analysis and drafted the manuscript. JY carried out the immunoassays. MJJ participated in the animal work, participated in the design of the study and reviewed the manuscript. DC performed the histological evaluation of samples and assisted in data analysis. RH conceived of the study, and participated in its design and coordination and helped to draft the manuscript. All authors read and approved the final manuscript.

## References

[B1] Heller LC, Heller R (2006). In vivo electroporation for gene therapy. Hum Gene Ther.

[B2] Favard C, Dean DS, Rols MP (2007). Electrotransfer as a non viral method of gene delivery. Curr Gene Ther.

[B3] Badea I, Verrall R, Baca-Estrada M, Tikoo S, Rosenberg A, Kumar P, Foldvari M (2005). In vivo cutaneous interferon-gamma gene delivery using novel dicationic (gemini) surfactant-plasmid complexes. J Gene Med.

[B4] Kim A, Lee EH, Choi SH, Kim CK (2004). In vitro and in vivo transfection efficiency of a novel ultradeformable cationic liposome. Biomaterials.

[B5] Meykadeh N, Mirmohammadsadegh A, Wang Z, Basner-Tschakarjan E, Hengge UR (2005). Topical application of plasmid DNA to mouse and human skin. J Mol Med.

[B6] Raghavachari N, Fahl WE (2002). Targeted gene delivery to skin cells in vivo: a comparative study of liposomes and polymers as delivery vehicles. J Pharm Sci.

[B7] Shi Z, Curiel DT, Tang DC (1999). DNA-based non-invasive vaccination onto the skin. Vaccine.

[B8] Vyas SP, Singh RP, Jain S, Mishra V, Mahor S, Singh P, Gupta PN, Rawat A, Dubey P (2005). Non-ionic surfactant based vesicles (niosomes) for non-invasive topical genetic immunization against hepatitis B. Int J Pharm.

[B9] Wang J, Hu JH, Li FQ, Liu GZ, Zhu QG, Liu JY, Ma HJ, Peng C, Si FG (2007). Strong cellular and humoral immune responses induced by transcutaneous immunization with HBsAg DNA-cationic deformable liposome complex. Exp Dermatol.

[B10] Alexander MY, Akhurst RJ (1995). Liposome-medicated gene transfer and expression via the skin. Hum Mol Genet.

[B11] Watabe S, Xin KQ, Ihata A, Liu LJ, Honsho A, Aoki I, Hamajima K, Wahren B, Okuda K (2001). Protection against influenza virus challenge by topical application of influenza DNA vaccine. Vaccine.

[B12] Yu WH, Kashani-Sabet M, Liggitt D, Moore D, Heath TD, Debs RJ (1999). Topical gene delivery to murine skin. J Invest Dermatol.

[B13] Liu PY, Wang XT, Badiavas E, Rieger-Christ K, Tang JB, Summerhayes I (2005). Enhancement of ischemic flap survival by prefabrication with transfer of exogenous PDGF gene. J Reconstr Microsurg.

[B14] (2000). Electrochemotherapy, Electrogenetherapy, and Transdermal Drug Delivery – Electrically Mediated Delivery of Molecules to Cells.

[B15] Titomirov AV, Sukharev S, Kistanova E (1991). In vivo electroporation and stable transformation of skin cells of newborn mice by plasmid DNA. Biochim Biophys Acta.

[B16] Glasspool-Malone J, Somiari S, Drabick JJ, Malone RW (2000). Efficient nonviral cutaneous transfection. Mol Ther.

[B17] Drabick JJ, Glasspool-Malone J, King A, Malone RW (2001). Cutaneous transfection and immune responses to intradermal nucleic acid vaccination are significantly enhanced by in vivo electropermeabilization. Mol Ther.

[B18] Maruyama H, Ataka K, Higuchi N, Sakamoto F, Gejyo F, Miyazaki J (2001). Skin-targeted gene transfer using in vivo electroporation. Gene Ther.

[B19] Dujardin N, Van Der SP, Preat V (2001). Topical gene transfer into rat skin using electroporation. Pharm Res.

[B20] Heller R, Schultz J, Lucas ML, Jaroszeski MJ, Heller LC, Gilbert RA, Moelling K, Nicolau C (2001). Intradermal delivery of interleukin-12 plasmid DNA by in vivo electroporation. DNA Cell Biol.

[B21] Chesnoy S, Huang L (2002). Enhanced cutaneous gene delivery following intradermal injection of naked DNA in a high ionic strength solution. Mol Ther.

[B22] Zhang L, Nolan E, Kreitschitz S, Rabussay DP (2002). Enhanced delivery of naked DNA to the skin by non-invasive in vivo electroporation. Biochimica et Biophysica Acta-General Subjects.

[B23] Babiuk S, Baca-Estrada ME, Foldvari M, Baizer L, Stout R, Storms M, Rabussay D, Widera G, Babiuk L (2003). Needle-free topical electroporation improves gene expression from plasmids administered in porcine skin. Mol Ther.

[B24] Marti G, Ferguson M, Wang J, Byrnes C, Dieb R, Qaiser R, Bonde P, Duncan MD, Harmon JW (2004). Electroporative transfection with KGF-1 DNA improves wound healing in a diabetic mouse model. Gene Ther.

[B25] Medi BM, Hoselton S, Marepalli RB, Singh J (2005). Skin targeted DNA vaccine delivery using electroporation in rabbits. I: efficacy. Int J Pharm.

[B26] Pavselj N, Preat V (2005). DNA electrotransfer into the skin using a combination of one high- and one low-voltage pulse. J Control Release.

[B27] Thanaketpaisarn O, Nishikawa M, Yamashita F, Hashida M (2005). Tissue-specific characteristics of in vivo electric gene: transfer by tissue and intravenous injection of plasmid DNA. Pharm Res.

[B28] Lin MP, Marti GP, Dieb R, Wang J, Ferguson M, Qaiser R, Bonde P, Duncan MD, Harmon JW (2006). Delivery of plasmid DNA expression vector for keratinocyte growth factor-1 using electroporation to improve cutaneous wound healing in a septic rat model. Wound Repair Regen.

[B29] Heller LC, Jaroszeski MJ, Coppola D, Mccray AN, Hickey J, Heller R (2007). Optimization of cutaneous electrically mediated plasmid DNA delivery using novel electrode. Gene Ther.

[B30] Vandermeulen G, Staes E, Vanderhaeghen ML, Bureau MF, Scherman D, Preat V (2007). Optimisation of intradermal DNA electrotransfer for immunisation. J Control Release.

[B31] Liu L, Marti GP, Wei X, Zhang X, Zhang H, Liu YV, Nastai M, Semenza GL, Harmon JW (2008). Age-dependent impairment of HIF-1alpha expression in diabetic mice: Correction with electroporation-facilitated gene therapy increases wound healing, angiogenesis, and circulating angiogenic cells. J Cell Physiol.

[B32] Hirao LA, Wu L, Khan AS, Satishchandran A, Draghia-Akli R, Weiner DB (2008). Intradermal/subcutaneous immunization by electroporation improves plasmid vaccine delivery and potency in pigs and rhesus macaques. Vaccine.

[B33] Gilbert RA, Jaroszeski MJ, Heller R (1997). Novel electrode designs for electrochemotherapy. Biochim Biophys Acta.

[B34] Sersa G, Cemazar M, Semrov D, Miklavcic D (1996). Changing electrode orientation improves the efficacy of electrochemotherapy of solid tumors in mice. Bioelectrochem Bioenerg.

[B35] Faurie C, Phez E, Golzio M, Vossen C, Lesbordes JC, Delteil C, Teissie J, Rols MP (2004). Effect of electric field vectoriality on electrically mediated gene delivery in mammalian cells. Biochim Biophys Acta.

[B36] Heller L, Jaroszeski MJ, Coppola D, Pottinger C, Gilbert R, Heller R (2000). Electrically mediated plasmid DNA delivery to hepatocellular carcinomas in vivo. Gene Therapy.

[B37] Wells JM, Li LH, Sen A, Jahreis GP, Hui SW (2000). Electroporation-enhanced gene delivery in mammary tumors. Gene Ther.

[B38] Cemazar M, Sersa G, Wilson J, Tozer GM, Hart SL, Grosel A, Dachs GU (2002). Effective gene transfer to solid tumors using different nonviral gene delivery techniques: electroporation, liposomes, and integrin-targeted vector. Cancer Gene Ther.

